# Immune Checkpoints and Innate Lymphoid Cells—New Avenues for Cancer Immunotherapy

**DOI:** 10.3390/cancers13235967

**Published:** 2021-11-27

**Authors:** Nicolas Jacquelot, Maryam Ghaedi, Kathrin Warner, Douglas C. Chung, Sarah Q. Crome, Pamela S. Ohashi

**Affiliations:** 1 Princess Margaret Cancer Centre, University Health Network, Toronto, ON M5G 2M9, Canada; maryam.ghaedi@uhnresearch.ca (M.G.); kathrin.warner@uhnresearch.ca (K.W.); doug.chung@mail.utoronto.ca (D.C.C.); 2 Department of Immunology, University of Toronto, Toronto, ON M5S 1A8, Canada; sarah.crome@utoronto.ca; 3 Ajmera Transplant Centre, Toronto General Hospital Research Institute, University Health Network, Toronto, ON M5G 2C4, Canada

**Keywords:** cancer, innate lymphoid cells, natural killer cells, immunotherapy, immune checkpoints, migration, trafficking, tissue homeostasis, immune regulation

## Abstract

**Simple Summary:**

Targeting the inhibitory receptors expressed by immune cells has revolutionized the clinical management of cancer patients. Initially developed to enhance T cell responses, recent investigations have demonstrated that innate lymphoid cells (ILC) also express many of these checkpoint molecules and could therefore be impacted by checkpoint blockade-based therapies. The diversity of the innate lymphoid cell family and their critical role in maintaining tissue homeostasis has drawn broad interest from the field to investigate their function in cancer. Here, we discuss recent findings highlighting the diversity of ILC in tissues and their capacity to migrate into organs upon inflammatory challenge. We further provide a comprehensive overview of the current knowledge on immune checkpoint (IC) expression on ILC, focusing on their therapeutic potential and capacity to modulate anti-tumor immune response.

**Abstract:**

Immune checkpoints (IC) are broadly characterized as inhibitory pathways that tightly regulate the activation of the immune system. These molecular “brakes” are centrally involved in the maintenance of immune self-tolerance and represent a key mechanism in avoiding autoimmunity and tissue destruction. Antibody-based therapies target these inhibitory molecules on T cells to improve their cytotoxic function, with unprecedented clinical efficacies for a number of malignancies. Many of these ICs are also expressed on innate lymphoid cells (ILC), drawing interest from the field to understand their function, impact for anti-tumor immunity and potential for immunotherapy. In this review, we highlight ILC specificities at different tissue sites and their migration potential upon inflammatory challenge. We further summarize the current understanding of IC molecules on ILC and discuss potential strategies for ILC modulation as part of a greater anti-cancer armamentarium.

## 1. Introduction

The appraisal of innate immune cell diversity has grown over the last decades, owing to the identification of many additional subsets. Particularly, the discovery of the family of innate lymphoid cells (ILC) as an independent lymphoid lineage devoid of antigen-specific T and B cell receptors has reshaped our view of the immune system [1,2]. The ILC family is currently classified into five subsets—namely NK cells, ILC1, ILC2, ILC3 and lymphoid tissue inducer (LTi) cells—that are considered the innate counterparts of the T lymphocytes, with the exception of LTi cells [3]. NK cells bear cytotoxic features as CD8 T cells and “helper” ILC1, ILC2 and ILC3 mirror CD4 T helper (h)1, Th2 and Th17/22 subsets [3]. LTi cells are critical to lymph node development during embryogenesis [4,5,6]. NK cells patrol throughout the body, whereas non-NK cell ILC are predominantly tissue resident. ILC maintain tissue homeostasis by rapid recognition and response to danger signals occurring during tissue injury or infection. A core transcriptional program specific to each ILC subset dictates their developmental pathway and function [3,7,8,9]. NK cells and ILC1 express the transcription factors Eomesodermin (EOMES) and T-box transcription factor 21 (T-BET), which drive the production of interferon (IFN)-γ and cytotoxic molecules such as granzymes and perforin, key features involved in the elimination of virus-infected and tumor cells [9,10,11]. ILC2 express the transcription factors GATA-binding protein 3 (GATA3) and retinoic acid-related orphan receptor α (ROR-α), which drive the secretion of interleukin (IL)-4, IL-5, IL-13 and granulocyte macrophage-colony stimulating factor (GM-CSF), and respond to allergens and extracellular parasites [12]. ILC3 express retinoic acid receptor-related orphan nuclear receptor γt (RORγt), produce IL-17A and IL-22 and have important functions in host defense against extracellular bacteria and fungi [13]. Recent evidence has shown that ILC also impact tissue repair and remodelling, host metabolism homeostasis and can communicate with the nervous system [3,14]. Collectively, these properties identify ILC as key regulators of the immune response and tissue homeostasis [15].

ILC are equipped with a wide range of surface and nuclear receptors, including cytokine and chemokine receptors, which integrate signals from their microenvironment to trigger their effector functions [14]. They also express major histocompatibility complex (MHC) Class II molecules, influencing the function and activity of CD4^+^ T cells [14]. In addition, they express a myriad of co-stimulatory and inhibitory receptors/ligands, including programmed cell death 1 (PD-1)/ligand (PD-L1), inducible T cell co-stimulator (ICOS)/ligand (ICOSL), glucocorticoid-induced tumor-necrosis-factor-receptor-related protein (GITR)/ligand (GITRL), killer cell lectin-like receptor G1 (KLRG1), T-cell immunoglobulin domain and mucin domain 3 (TIM-3), lymphocyte-activation gene 3 (LAG-3), B and T lymphocyte attenuator (BTLA), cytotoxic T-lymphocyte antigen-4 (CTLA-4) and OX40 ligand (OX40L), which further regulate their functions [14]. These ICs control ILC function at both steady state and during inflammation influencing self-tolerance and immunity. Thus, a growing interest in the field is to comprehensively understand the role of IC on ILC function to modulate their activities in health and disease.

In this review, we delineate the key roles of ILC in tissue homeostasis, immune tolerance and tumor immunity, with a specific focus on the expression pattern and the function of IC on ILC and potential for therapeutic interventions.

## 2. Innate Lymphoid Cell Diversity within Tissues

The proportion of ILC subsets differs according to the organ [16]. ILC transcriptional profile, phenotype and function are heavily influenced by their microenvironment and their phenotypes vary largely from tissue to tissue [16,17,18,19,20,21,22,23]. Genetic fate-mapping in mouse using key ILC features has further confirmed that ILC exhibit substantial plasticity and can modify their phenotype and function to resemble other ILC subsets, depending on the microenvironment signals [23,24,25,26]. Understanding how tissue signals impact ILC transcriptional profiles, phenotype, and functions is critical for the development of therapies that can target ILC in a subset and tissue-dependent manner.

In both mice and humans, NK cells represent the most abundant ILC subset, widely distributed across all tissues and organs [27,28], and can exhibit tissue-adapted phenotypes and functions [10,29,30]. Immature human NK cells are preferentially found in lymph nodes and intestines, whereas more mature NK cells localise in blood, bone marrow, spleen and lungs [30]. Notably, organ-specific gene signatures of NK cells are conserved between mice and humans [29].

In mice, while ILC1 are mainly found in liver, ILC2 and ILC3 are highly represented in mucosal barriers and metabolic tissues [27,31,32,33]. In humans, however, ILC1 and ILC3 are the most-represented CD127^+^ ILC subsets in most tissues, whereas ILC2 predominantly locate in abdominal fat and lungs, where they represent 20–30% of CD127^+^ ILC [27]. Human CD127^+^ ILC isolated from blood, tonsils, lungs and the colon were shown to exhibit a tissue-specific transcriptional imprinting. Blood and tonsil ILC clustered closely and separated from other tissue-resident ILC, irrespective of the ILC ontogeny [34]. Blood and tonsil ILC expressed protein-coding genes, including *SELL*, *SIPR1*, *ICAM3*, *ITGB2*, *ITGAL*, *ITGAM* and *S100*, involved in cell adhesion and migration. In contrast, tissue-resident ILC, and to some extent tonsil ILC, expressed higher levels of *CD69*, *AREG*, *CD44*, *NR4A1*, as well as transcripts implicated in the Fos/Jun and NFκB signalling [34].

In mice, ILC1 were identified as a distinct ILC lineage, based on their unique transcription factor expression and developmental trajectory [35,36]. ILC1 undergo a tissue- specific transcriptional imprinting. Hence, they differentially express transcription factors and surface molecules including *Zfp683* (HOBIT), *Il7r*, *Cd200r1*, *Itga1* (CD49a), *Itgae* (CD103), *Cd69* and *Cxcr6*, according to their specific tissue [9,37]. As an example, *Hobit* deficiency impaired ILC1 development in liver but not in other organs [36]. In humans, ILC1 were mainly found in secondary lymphoid organs, salivary glands and the liver [27,38,39]. However, it is notable that human ILC1 lack a specific marker expression profile that distinguishes them from other ILC, rendering their identification complicated [34,40,41].

Transcriptional profiling of ILC2 at single cell and bulk levels from different tissues has indicated that these cells harbour a high degree of transcriptional imprinting by tissue microenvironment signals [16]. ILC2 homeostasis and function is differentially regulated by thymic stromal lymphopoietin (TSLP), IL-33, IL-25, IL-18, IL-7, IL-2 and IL-1β, depending on the tissue [14]. Lung and fat ILC2 are more dependent on IL-33 signaling for their activation, while gut and skin ILC2 are more dependent on IL-25 and IL-18 signaling, respectively. However, ILC2 tissue-dependent transcriptional imprinting occurs independently of TSLP, IL-33 and IL-25 signaling [16]. Consistently, upon adoptive transfer of ILC2 into lymphocyte-deficient *Rag2^−/−^Il2rg^−/−^* mice, their phenotype was shaped by the new tissue microenvironment signals, regardless of their tissue of origin [42]. In humans, blood and tonsil ILC2 were shown to express a high level of transcripts involved in cell trafficking and migration, such as *CRTH2*, *S1PR2* and *CCR2* [34]. In contrast, tissue resident ILC2 highly expressed genes involved in their activation, including *IL1RL1* (IL-33 receptor) and *IL17RB* (IL-25 receptor), as well as genes involved in lipid metabolism, such as *PPARG*, *HPGD*, *HPGDS* and *MBOAT2* [34]. In addition, lung resident ILC2 expressed higher levels of *IL13* transcripts than blood ILC2 [34]. Together, these results indicate that lung resident cells harbour a more activated phenotype than circulating ILC2. It is notable that while CD127 and CRTH2 are considered universal markers for identifying human ILC2, they are both downregulated upon receiving pro-inflammatory signals such as TSLP, IL-25, IL-33 or IL-2, suggesting that ILC2 frequencies might be underestimated in inflamed tissues [34,40,43]. Other markers, including NKp30, CD69, ICOS, CD122 or CD49a, are also differentially expressed on ILC2 across different tissues, with increased heterogeneity observed under inflammation and pathological conditions [40]. Collectively, these findings support a central role for tissue-specific signals in the ILC2 transcriptional profile, phenotype and function, which might underlie the differential pro- or anti-tumorigenic activities of ILC2, depending on the specific tumor type [44,45,46,47,48,49].

Mouse ILC3 also show great heterogeneity in their phenotype and function depending on their tissue [13]. Mouse ILC3 have been observed in the spleen, skin, lungs, gut, peripheral and mLNs [13,17,31,46]. Intestinal ILC3 can be subdivided into three major subsets based on the expression of NKp46 and CCR6, namely NKp46^−^CCR6^+^ LTi-like, NKp46^−^CCR6^−^ and NKp46^+^CCR6^−^ subsets. NKp46 upregulation in CCR6^−^ ILC3 is driven by T-bet, which is progressively expressed upon stimulation with pro-inflammatory signals from gut microbiota [50]. More recently, T-bet expression was shown to be regulated by c-Maf and Notch signalling [22,51,52,53,54,55], in line with a complex regulatory network modulating the phenotype and function of ILC3. In accordance, cytokines, including IL-1β, TGF-β, IL-12, IL-15, IL-18 and IL-23, impact ILC3 plasticity toward inflammatory NKp46^+^ ILC3 as well as ILC1, ILC2 or regulatory ILC-like cells [23]. The adoptive transfer of ex-ILC3 into *Rag2^−/−^Il2rg^−/−^* mice has demonstrated that these cells are also imprinted by the tissue in which they traffic to, irrespective of their tissue of origin [26], an observation further confirmed using human ILC3 [56]. Interestingly, splenic ex-ILC3 displayed an inflammatory ILC1/NK cell-like phenotype, and their adoptive transfer in tumor-bearing mice suppressed tumor growth in an IL-12 dependent manner [26]. In humans, in vitro and in vivo analyses have revealed the capacity of lineage (CD3, CD19, CD34, CD123, CD303, FcεRI)^−^CD127^+^c-kit^+^CRTH2^−^ cells, originally defined as ILC3, to give rise to multiple mature ILC [57]. Based on these observations, this subset has been since reclassified as ILC progenitors (ILCp). ILCps are divided based on the expression of KLRG1 and NKp46. KLRG1^+^ ILCp predominantly differentiate into ILC2, whereas NKp46^+^ ILCp mainly differentiate into ILC3 [58]. These ILCps are also present in tissues, and may differentiate into mature ILC in response to local microenvironment signals. In tissues, lineage^−^CD127^+^c-kit^+^CRTH2^−^ cells that also express NKp44 represent *bona fide* ILC3. Human ILC3 are also heavily imprinted by their tissue environment. Lung and colon ILC3 were shown to express unique tissue residency features, including *CSF2* (GM-CSF) *BHLHE40*, *CXCL8* and *VEGFA* [34]. In addition, lung and colon ILC3 expressed higher levels of HLA-DP, -DQ and -DR genes compared to tonsil, whereas tonsil and colon ILC3 expressed higher levels of *NCR2* (NKp44), *TNFSF13B* (BAFF) and *CD300LF* than lung [34]. ILC3 in colon preferentially expressed high levels of *KIT*, *IL1R1*, *TNFSF11* (RANKL), *TNFSF4* (OX40L), *ICOS*, *CCR6*, *AHR* and *LIF* [13,34]. Interestingly, a gradient of ILC3-ILC1-like cells has been described in human tonsils and intestinal lamina propria, identified based on the expression of CD103, CD300LF and CCR6 [56], a phenotype driven by microenvironment signals [34,56]. Collectively, these studies have demonstrated a high degree of ILC3 heterogeneity and plasticity according to tissue type and microenvironment signals. Hence, a better understanding of ILC3 heterogeneity and plasticity in tumor development, progression, prognosis and treatment responses is needed.

## 3. In Situ ILC-Poiesis and Interorgan ILC Migration

Tissue resident ILC act as local gatekeepers to maintain immunological balance and tissue homeostasis. Although tissue compartmentalization occurs at steady state [59,60,61,62,63], non-NK cell ILC are able to traffic between organs in a tissue and subset-dependent manner upon inflammatory perturbations [61,62,64,65,66,67,68,69,70]. This ability of ILC to migrate to other organs ensures that appropriate immune cells are distributed properly to constrain insults and tissue damage within the body. In addition to ILC migration, ILC develop from progenitors in multiple compartments, including bone marrow, fetal liver and peripheral tissues to assure the constant pool of diverse ILC within tissues during steady state and inflammation [7,35,69,71,72].

Circulating and tissue-resident NK cells display differential expression profiles of adhesion molecules and chemokine receptors, including CCR5, CCR7, CD62L, CXCR3, CXCR4, CXCR5, CXCR6, CX3CR1, α4β7, S1PR5, CD69 and CD103, which, together, dictate their ability to egress from bone marrow or lymph nodes and to migrate within and between organs [28,29,73,74,75]. While NK cell trafficking has been extensively studied, we know much less about the mechanisms involved in non-NK cell ILC migration. A handful of studies suggests that mouse ILC migrate between lymphoid and non-lymphoid organs, particularly under inflammatory conditions.

**Figure 1:**
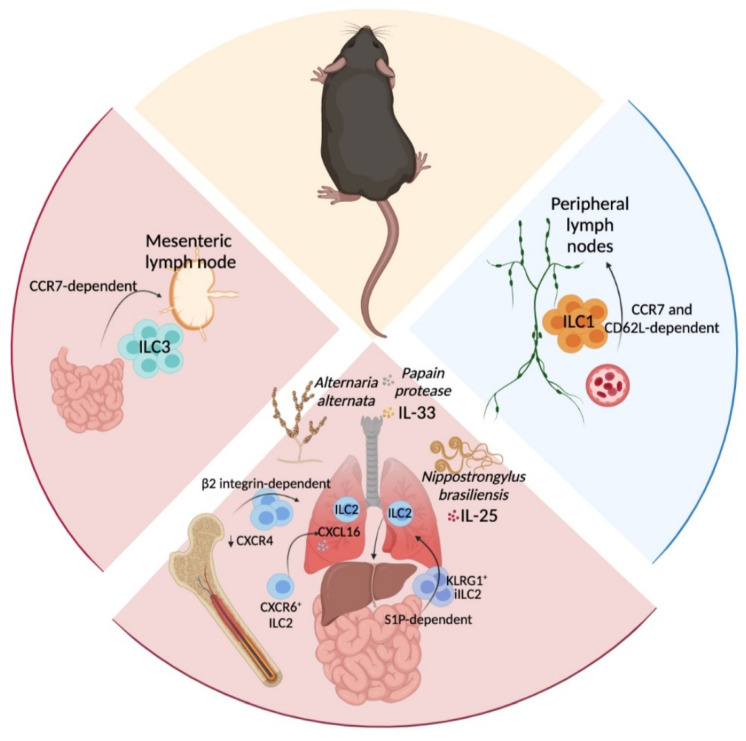
Murine ILC migrate interorgans under physiological and pathogenic conditions. The above schematic representation summarizes ILC trafficking pathways. At steady state, ILC1 migrate to the peripheral lymph nodes using the blood stream in a CCR7 and CD62L-dependent manner. In contrast, ILC2 and ILC3 traffic to lymph or blood stream following inflammatory insults. ILC2 migrate from bone marrow and small intestine to populate the lung. In addition, lung ILC2 migrate to the liver. Intestinal ILC3 mainly traffic from and to mLN to promote a local inflammatory immune response. This figure has been created with BioRender.com.

At steady state, most of the migratory ILC in peripheral lymph nodes are ILC1, whereas ILC2 and ILC3 are mainly resident cells. ILC1 enter peripheral lymph nodes from circulation in a CCR7- and CD62L-dependent manner [62] (Figure 1).

During inflammatory conditions, ILC2 are mobilized from bone marrow and tissues using different migratory patterns [65,76,77] (Figure 1). IL-33- or *Alternaria*-induced lung inflammation promoted the downregulation of the chemokine receptor C-X-C chemokine receptor (CXCR)4 on IL-33R-expressing ILC2 in bone marrow and their migration to the lung [65], in a β2 integrin-dependent manner [77] (Figure 1). Irradiation of one animal in parabiotic mice [65] induced ILC2 migration from one parabiotic partner to the lungs, skin and mesenteric lymph nodes (mLN) of the other. In this case, ILC2 migration to the lung and mLN, but not the skin, was further amplified by combining irradiation with systemic IL-33 injections [65]. Supporting this, intranasal IL-33 injection resulted in increased C-X-C chemokine ligand (CXCL)16 expression in the lungs [66], which led to the migration of CXCR6^+^ ILC2 to the lungs and mediastinal lymph nodes [66]. In addition, IL-25 treatment or *Nippostrongylus brasiliensis* infection induced migration of KLRG1^+^ inflammatory (iILC2) from small intestine to lymphatic system in a sphingosine 1 phosphate (S1P)-dependent manner and eventually to the lungs [64]. Another study has revealed that blood ILC2 that emerge following *Nippostrongylus brasiliensis* infection display heterogenous phenotypes, and originate from different tissue niches [67]. Early post-infection blood ILC2 were mainly derived from the small intestine and these cells were progressively replaced by lung-derived ILC2 during the course of infection. In addition, intranasal administrations of IL-33 or papain promoted ILC2 migration through the blood stream to the liver [70]. These findings indicate that local tissue perturbations induce systemic ILC2 migration and type 2 inflammation.

Intestinal ILC migrate to the draining lymph nodes, where they localise in the interfollicular areas, zones of T cell priming, to potentially influence the generation of adaptive immune responses [78]. The migration of ILC3 to the mLN was shown to be dependent on C-C chemokine receptor (CCR)7 expression (Figure 1). Migratory ILC in lymphatic vessels have increased expression of genes involved in cell migration such as *S1pr1*, *S1pr4*, *Ccr7*, *Ccr10* or *Sell*, and decreased expression of genes involved in tissue retention, such as *Cxcr6* or *CD69*, compared to tissue resident ILC [68]. Intestinal infection with *Salmonella typhimurium* does not increase the frequency or number of migrating ILC to the mLN, but modifies the composition of migrating ILC in lymphatic vessels towards increased ILC3 frequency [68]. These migratory ILC from infected animals have increased expression of interferon response genes, pro-inflammatory chemokine genes such as *Ccl3* or *Cxcl10* and cytokines IL-22, IFN-γ and GM-CSF compared to migratory ILC from control mice [68].

In humans, non-NK cell ILC are found both in tissues and circulation. In addition, a systemic ILC precursor, which can give rise to NK cells, ILC1, ILC2 and IL-22-expressing (but not IL-17A-expressing) ILC3 is also found both in tissues and circulation. These cells have the ability to differentiate into ILC within tissues in response to the tissue microenvironment signals during homeostatic and inflammatory conditions [57,79]. CRTH2 expression by human ILC2 [80] results in sensitivity to the lipid mediator prostaglandin E2 [81] and regulates ILC2 migration [82]. Additionally, chemokine receptors and integrins expressed at the transcriptional or protein level on mouse/human ILC have been identified. These include the chemokine receptors *Ccr2*, *Ccr4*/CCR4, *CCR6*/CCR6, *CCR7*/CCR7, CCR8, CCR9, CCR10, *CXCR4*, CXCR3 and *CXCR5 CXCR6*/CXCR6, the integrins *Icam1*, *ITGB2*, *ITGAM*, *ITGAL*, *ICAM3*, α4β7, αLβ2, α4β1 and *S1PR1* and the selectins cutaneous lymphocyte antigen (CLA) or *SELL*/CD62L [34,42,49,58,61,62,63,77,83,84]. These observations further highlight that specific patterns of chemokine receptors, integrins and selectins expression enable mouse and human ILC to appropriately traffic within and between tissues to mediate their effector function [61,83].

## 4. Peripheral Immune Tolerance—The Role of Immune Checkpoints

Immune cells must distinguish between mounting an appropriate immune response to an immunological threat and self-reactivity, engendering tissue damage and autoimmunity. In addition to central tolerance mechanisms that eliminate most autoreactive T cells, peripheral tolerance mechanisms limit potential autoreactive immune cells. These include regulatory adaptive and innate immune cells as well as IC signalling pathways such as PD-1 or CTLA-4 [85,86,87,88,89]. PD-1 deficiency results in spontaneous development of autoimmune disease and the disease onset is accelerated in autoimmune-prone backgrounds [90]. On a C57BL/6 background, aged mice deficient in PD-1 develop characteristic lupus-like proliferative arthritis and glomerulonephritis [91]. On a BALB/c background, PD-1 deficient mice develop fatal dilated cardiomyopathy with large thrombosis due to the diffuse deposition of autoantibodies on the surface of cardiomyocytes [92,93]. The phenotype of CTLA-4 deficient mice is even more severe, with mice dying from lymphoproliferative syndrome and fatal multiorgan destruction at 4 weeks of age due to defects in Tregs and the absence of key T effector inhibitory pathways [94,95]. In humans, PD-1 deficiency and germline mutations in *CTLA-4* increase the susceptibility to infections and autoimmune disorders [96,97,98,99]. Together, these mouse models and clinical observations have demonstrated the fine balance between immune cell activation and tolerance, governing immune system equilibrium and tissue homeostasis [100,101].

## 5. ILC and Immune Checkpoints. What Do We Know?

In cancer, immune inhibition due to expression of IC by tumor-infiltrating T cells has promoted the development of immunotherapies to specifically block these pathways [102,103] and has led to major breakthroughs in cancer treatment [104,105,106,107,108,109,110]. Over the past decade, IC expression by ILC has been widely reported. However, we are only beginning to understand the role of these molecules in regulating ILC function in health and disease. It appears that similarities between ILC and adaptive T cells involve akin regulatory pathways [111]. Consequently, the analysis of IC expression and function in ILC, with implications for ILC-mediated tissue homeostasis, pathogen clearance and anti-tumor responses has recently attracted vast interest.

## 6. ILC Expression of IC—Parallel with Adaptive Immune Cells

**Figure 2:**
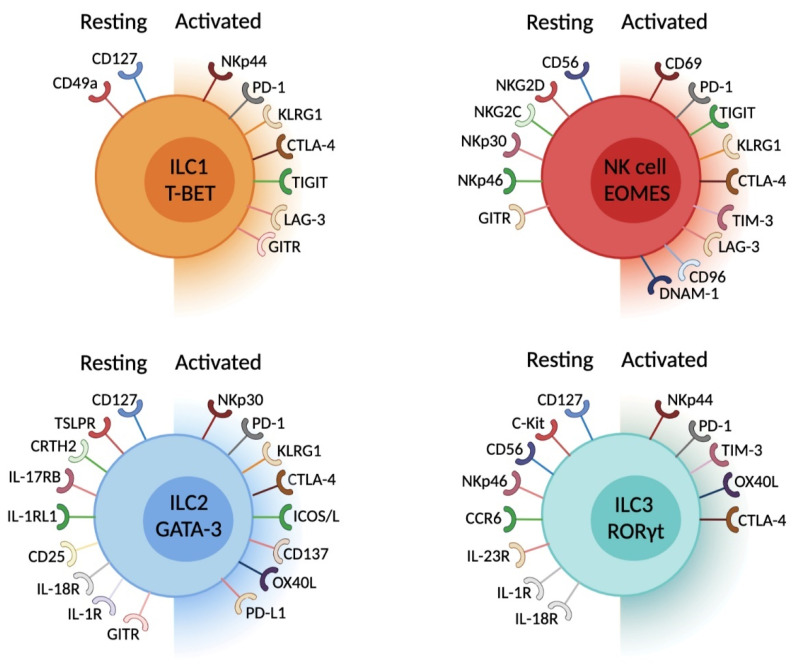
Cytokine and chemokine receptors and IC expression on mouse or human ILC according to their activation status. This figure has been created with BioRender.com.

Tissue-resident ILC express, either constitutively or under specific inflammatory conditions, a myriad of stimulatory and inhibitory receptors. These include PD(L)-1, CTLA-4, TIGIT, DNAM-1, CD96, BTLA, NKp44, NKp46, ICOS(L), KLRG1, OX40L, GITR(L), TIM-3 and LAG-3 (Table 1 and Figure 2). The role and impact of these IC on adaptive lymphocytes have been extensively characterized [112,113]. Herein, we will review the current understanding of the contribution of IC molecules on ILC function in health and disease (Table 1).

### 6.1. PD-1 and Its Ligands

PD-1 is an inhibitory receptor originally identified on dying activated T cells [205]. Three decades of intensive efforts to comprehensively characterize this new member of the immunoglobulin gene superfamily has resulted in (i) the discovery of PD-L1 [206,207] and PD-L2 [208,209], two PD-1 ligands that are widely expressed across tissues and cell populations [86,88,101], (ii) the characterization of PD-1 inhibitory function on T cells in chronic infection and cancer [102,103,210], (iii) better understanding of the role of PD-1 in autoimmunity [91,92], and (iv) the appraisal of widespread PD-1 expression across many immune cell types [101,162,211,212].

#### 6.1.1. PD-1 Expression in ILC Development

A proportion of ILC bone marrow progenitors (Lin^−^α4β7^+^IL-7Rα^+^Flt3^−^PLZF^+^) expressed PD-1 [162,212]. The adoptive transfer of PD-1^+^ ILCp into *Rag2*^−/−^*Il2rg*^−/−^ mice, mainly, has given rise to non-NK ILC1, ILC2 and ILC3 [162], identifying PD-1 expression as a marker to efficiently isolate mouse ILCp. Despite these observations, PD-1 deficiency does not appear to affect the number of ILCp in bone marrow or their development into mature ILC at steady state [170,212].

#### 6.1.2. PD-1 and NK Cells

At steady state, mouse NK cells do not express PD-1 [115,162] (Figure 2). However, PD-1 is expressed on tumor-infiltrating NK cells, identifying more responsive cells compared with their PD-1^−^ counterparts [115]. The binding of PD-1 to PD-L1 expressed by tumor cells induced NK cell dysfunction and tumor escape [115]. In vivo blockade of PD-1 resulted in improved anti-tumor responses in an NK cell-dependent manner. PD-1 expression has been detected on human NK cells from both healthy individuals [117,118] and cancer patients [117,119,120,121,122,123,124,131]. The analysis of 200 healthy donors [117] has shown that approximatively 25% of individuals harbour PD-1-expressing circulating NK cells, an expression restricted to the CD56^dim^ subset. The induction of PD-1 expression on human NK cells was dependent on pro-inflammatory cytokines, the engagement of activating receptors and the levels of glucocorticoids [116,119,122]. Increased PD-1 expression was observed on NK cells isolated from ascites of ovarian carcinoma patients [117], suggesting that, similar to T cells, PD-1 expression on NK cells may be induced upon stimulation. Similar findings were reported in Kaposi sarcoma [119], Hodgkin lymphoma [120], digestive [123,124], lung [122] and breast [124] cancers as well as pleural effusions of primary and metastatic tumors [121]. PD-1 expression [119] was also upregulated on NK cells from HIV and HCV-infected patients compared with non-infected individuals. Functional analyses of these cells have shown that PD-1^+^ NK cells displayed reduced cytotoxicity and cytokine production compared with PD-1^−^ NK cells, a phenotype that is reversible with anti-PD-1/PD-L1/2 blocking antibodies or upon stimulation with IL-2 and IL-15 [117,118,119,120]. Interestingly, recent reports have shown that mouse and human NK cells do not express PD-1 at the transcriptional levels but instead acquire surface PD-1 expression by trogocytosis [213], a process in which a part of a cell membrane is exchanged between two cell types during physical interactions. These results indicate that PD-1 expression is restricted to mature and activated NK cells, and inhibits NK cell function (Figure 2). Together, these findings suggest that PD-1 likely influences NK cell function and response to immunotherapy.

#### 6.1.3. PD-1 and ILC1

At steady state, mouse ILC1 express low levels of PD-1 [162] while tumor-infiltrating ILC1-like transdifferentiated NK cells express higher levels of PD-1 [161]. In addition, blood and tumor-infiltrating human ILC1 were shown to express PD-1/*PDCD1* [111]. These include non-small cell lung, breast and gastrointestinal tumor-infiltrating ILC1 [124,163].

#### 6.1.4. PD-1 and ILC2

Approximatively 20–40% of lung ILC2 express PD-1, a proportion that is further increased upon influenza infection or papain challenge [162]. We, and others, have shown that IL-33 and γc cytokines also induce PD-1 expression [44,46,170,179,180]. PD-1 is mainly expressed by KLRG1^+^ ILC2 [170] and negatively regulates ILC2 proliferation and cytokine expression [44,46,124,163,170,178,179,181,214] (Figure 2). PD-1-deficient mice (*Pdcd1^−/−^*) have increased peripheral ILC2 [46,170], with enhanced KLRG1 expression and cytokine production [170] compared with wildtype animals. Furthermore, PD-1 deficient ILC2 displayed a metabolic shift towards glycolysis, glutaminolysis and methionine catabolism, which is associated with increased effector function and survival [179]. Interestingly, blocking PD-1 or its signalling pathway SHP1/2 significantly increased the phosphorylation of STAT5 [170], suggesting that PD-1 negatively regulates the cytokine signalling pathways by limiting the phosphorylation of the STAT proteins, similar to mechanisms described for T cells [215,216]. In humans, circulating PD-1^+^ ILC2 from healthy donors displayed a decrease in cytokine expression and proliferation compared with their PD-1^−^ counterparts [170], indicating that PD-1 engagement potentially regulates similar pathways between mouse and human ILC2. A growing body of work indicates that tumor-infiltrating ILC2 express high levels of PD-1, which regulates ILC2 function and cancer prognosis [44,46,49,124,163,178]. Increased PD-1 expression was reported on ILC2 in inflammation-induced colorectal tumors as tumor development progresses. The authors suggest that these PD-1-expressing ILC2 are involved in colorectal cancer growth by facilitating cancer cell proliferation [178]. The analysis of PBMCs, peri-lesions and tumor lesions from breast and gastrointestinal cancer patients has revealed increased levels of PD-1 expression on tumor-infiltrating ILC2 compared with non-tumoral control tissues [124]. In NSCLC, ILC2 accumulated in tumor lesions and expressed higher levels of PD-1 than circulating ILC2 [163]. This phenotype was associated with increased IL-4 and IL-13 expression which, together, promoted macrophage M2 polarization [163]. These results suggest that ILC2 may contribute to the tumor immunosuppressive environment, promoting tumor growth and progression. Further observations in bladder cancer, prostate carcinoma and acute promyelocytic leukaemia supported a tumor-promoting function of ILC2 in these cancers [47,48]. In contrast, we and others, have described an improved anti-tumor immunity associated with ILC2 tumor infiltration and enhanced treatment responses in melanoma [46] and pancreatic adenocarcinoma [44]. In the latter, ILC2 sustained CD8^+^ T cell and dendritic cells tumor infiltration through an IL-33 and CCL5-dependent mechanism [44]. In melanoma, however, ILC2-derived GM-CSF expression promoted eosinophil tumor infiltration and survival associated with increased anti-tumor immunity [46]. In both cancer types, tumor-infiltrating ILC2 expressed high levels of PD-1, which dampened ILC2-dependent anti-tumor responses [44,46]. Combined IL-33 treatment with PD-1 blockade promoted ILC2 anti-tumor responses and resulted in reduced tumor size and prolonged mouse survival [44,46]. Collectively, these findings indicate that PD-1 expression on ILC2 inhibits their function, and the blockade of PD-1 using targeting agents amplifies the activity of these cells. However, how anti-PD-1 therapeutic antibodies influence ILC2 function in human cancers requires further investigation and may be influenced by the local microenvironment.

Independent studies found that ILC2 also expressed PD-L1/*CD274* [111,182]. In mice, PD-L1 expression was augmented upon *Nippostrongylus brasiliensis* infection [182] but not IL-33 injections [179]. PD-L1-expressing ILC2 interacted with PD-1-expressing T cells and promoted Th2 polarization and type 2 inflammatory responses [182]. PD-L1^+^ ILC2 may influence the function and activity of PD-1^+^ ILC2 either by interacting in cis or in trans, but the mechanism remains to be demonstrated.

#### 6.1.5. PD-1 and ILC3

PD-1 expression on ILC3 was reported in mouse intestine [162], human decidua [199] and cancer [49,121,124] (Figure 2). In breast and gastrointestinal tumors, tumor-infiltrating ILC3 expressed variable levels of PD-1 [124]. CD56^+^ ILC3 expressed low levels of PD-1 in pleural effusions of primary and metastatic tumors [121]. NCR (NKp44 and NKp46) stimulation of human decidua increased the expression of IL-22, whereas engagement of both NCR and PD-1 receptors reduced ILC3-derived IL-22, TNF-α and IL-8 expression [199], indicating that PD-1 inhibits ILC3 function. Collectively, these results suggest that PD-1 negatively regulates ILC3 function similarly to other ILC family members. Findings to date indicate that PD-1-targeting therapies would impact ILC3 effector function in cancer, which could in turn influence immunotherapy efficacies and clinical outcomes.

### 6.2. CTLA-4—CD80/CD86

At the immunological synapse, T cells typically engage CD28, a co-stimulatory receptor which binds to CD80 and CD86 at the surface of antigen-presenting cells [217,218]. Upon activation, T cells express CTLA-4 at their cell membrane, localizing at sites of TCR engagement [219]. CTLA-4 is a CD28 homolog, and can bind CD80 and CD86 with greater affinity than CD28 [164,165,166,167], thus acting as a negative regulator of T-cell activation [220]. CTLA-4 is also constitutively expressed by regulatory T cells and participates in their suppressive function [221,222].

Increasing evidence suggest that CTLA-4 is also expressed by multiple ILC populations under distinct conditions (Figure 2). Variable levels of CTLA-4 expression were detected in circulating and tumor-infiltrating NK cells and non-NK cell ILC in melanoma [160], hepatocellular carcinoma [49], breast and gastrointestinal [124] cancers, with higher levels found in tumor-infiltrating ILC compared to circulating cells. These findings suggest that specific signals from the tumor microenvironment may drive CTLA-4 expression in ILC and potentially regulate their function. In several mouse tumor models, the transdifferentiation of NK cells into intermediate and ILC1-like cells was accompanied by an increase in CTLA-4 expression [161]. This was further confirmed in human ILC1 [111]. At steady state, human circulating ILC2 and mouse intestinal ILC3 express CTLA-4 [37,124]. IL-10-producing ILC2 (so called ILC2_10_) with immunoregulatory potential, which are induced upon retinoic acid stimulation, have increased CTLA-4, PD-L1 and CD25 expression [183]. Upon ipilimumab (anti-CTLA-4 blocking antibody) treatment in melanoma patients, a significant decrease in NK cell, ILC1 and ILC2 frequencies has been observed, associated with reduced expression of the inhibitory receptor CD96 [160]. While no difference in the frequency of circulating ILC3 was reported, a reduced expression of CTLA-4 and NKp46 on these cells was noted [160]. The authors further showed that patients with disease control exhibited lower percentages of ILC1 compared with patients without disease control. Collectively, these results suggest that ipilimumab can modulate ILC frequency and phenotype, which may influence treatment efficacy and clinical outcomes. Further studies are needed to characterize the tissue signals that modulate CTLA-4 expression by ILC and how anti-CTLA-4 antibodies impact their function in cancer.

### 6.3. TIM-3

TIM-3 (CD366 or HAVCR2) is expressed at the surface of immune cells, regulating their function [223]. Four TIM-3 ligands have been described to date, namely Galectin-9, phosphatidylserine, high mobility group box 1 (HMGB1) and carcinoembryonic antigen cell adhesion molecule 1 (Caecam-1) [223]. TIM-3 is expressed by many immune cell types, including conventional and regulatory T cells, myeloid cells, dendritic cells, mast cells, NK cells and ILC3 [199,223]. At steady state, a high proportion of NK cells express TIM-3 [125,126] (Figure 2). Higher TIM-3 expression is found on CD56^dim^ NK cells than CD56^bright^ NK cell subset. NK cell stimulation with the cytokines IL-2, IL-12 and IL-18 or IL-15 further increase their TIM-3 expression [125,126], suggesting that similar to T cells, TIM-3 is upregulated upon NK cell activation (Figure 2). In accordance with this, high TIM-3 expression on NK cells is associated with highest IFN-γ production [125,126]. However, TIM-3 cross-linking impairs NK cell-derived IFN-γ expression associated with reduced killing capacities [125,126], indicating that TIM-3 negatively regulates NK cell function. TIM-3-mediated NK cell functional dysregulation in melanoma can be reversed by blocking TIM-3 [127]. High frequencies of TIM-3^+^ NK cells have been observed in many cancers. These include gastrointestinal stromal tumors [128], gastric cancer [129], oesophageal cancer [133], melanoma [127], colorectal cancer [130], anaplastic thyroid cancer [131], bladder cancer [132], hepatocellular carcinoma [134] and lung adenocarcinoma [135]. Higher TIM-3 levels are observed in most advanced stages of gastric cancer [129], and high levels of TIM-3 expression on NK cells correlated with reduced overall survival in oesophageal cancer, melanoma, hepatocellular carcinoma and lung adenocarcinoma [127,133,134,135]. In contrast, the frequency of circulating TIM-3^+^ NK cells in colorectal cancer was inversely correlated with clinical stages [130], suggesting a positive outcome associated with TIM-3-expressing NK cells in this setting. Collectively, these compelling findings indicate that TIM-3 negatively regulates NK cell function and anti-tumor responses in cancer. Human blood ILC express comparable levels *HAVCR2* to that of helper T cells [111]. About 40–60% of ILC3 isolated from human decidua expressed TIM-3 [199] (Figure 2). Most TIM-3^+^ ILC3 did not express PD-1 [199], suggesting that TIM-3 and PD-1 may identify two separate ILC3 subsets with distinct properties. NCR stimulation of single cell tissue preparations increased ILC3-derived IL-22 production, a stimulatory effect that is abrogated when TIM-3 was cross-linked [199]. Collectively, these results suggest that TIM-3 inhibits NK cell function, negatively influencing anti-tumor responses and cancer patients’ prognosis. However, the role and function of TIM-3 in non-NK cell ILC remains to be fully elucidated in tumors.

### 6.4. TIGIT, DNAM-1 and CD96

TIGIT is an inhibitory receptor belonging to a group of immunoglobulin superfamily receptors including DNAM-1 and CD96. These three receptors bind to CD155 (PVR), while TIGIT and DNAM-1 additionally engage CD112 (Nectin-2). DNAM-1 stimulates immune cell activity [138], whereas the binding of CD155 to CD96 or TIGIT negatively regulates immune cell function [139,140,141]. TIGIT is expressed by NK cells, ILC1 and ILC3 (Figure 2). Tumor-infiltrating NK cells expressed high levels of TIGIT, and TIGIT^+^ NK cells displayed impaired activity associated with increased expression of the inhibitory receptors TIM-3 and LAG-3 in comparison with TIGIT^−^ NK cells [142]. Full or conditional (specific deletion in NKp46-expressing cells, including NK cells) *TIGIT* deletion improved anti-tumor responses and survival of tumor-bearing mice compared to the wildtype group [142]. Similarly, the blockade of TIGIT using antibodies unleashed NK cell and T cell anti-tumor function [142]. The overall survival of tumor-bearing mice has been further increased following treatment with both anti-TIGIT and anti-PD-L1 antibodies [142], suggesting that non-redundant mechanisms exist between these two pathways. The analyses of circulating ILC isolated from healthy donors and melanoma patients has further confirmed that CD56^dim^ NK cells express TIGIT [160]. In contrast to colorectal cancer in which tumor-infiltrating NK cells displayed higher TIGIT expression than peri-tumoral NK cells [142], no major difference in TIGIT expression was noted on circulating NK cells between healthy donors and melanoma patients [160]. This study has also indicated that circulating ILC3, but not other ILC subsets [160], expressed TIGIT. Another study found that circulating ILC1 and to a lesser extent ILC2, but not ILCp, expressed *TIGIT* [111].

NK cell conversion into intermediate ILC1 or ILC1-like cells in the presence of TGF-β was associated with increased CD96 together with reduced DNAM-1 expression in mouse tumor models [161], likely driving the observed impairment of the anti-tumor immune response. Indeed, CD96 competes with DNAM-1 for CD155 binding, which limits NK cell function [140]. Complete loss of *Cd96* expression or therapeutic blockade of CD96 using antibodies enhanced NK cell hyperresponsiveness, improved protection against B16F10 lung metastases and reduced the development of chemically-induced tumors [140,224]. Interestingly, mouse ILC progenitors had high DNAM-1 levels, particularly the ILC2 progenitors [212], which potentially explains why mature ILC2 express this receptor. Human circulating ILC2, but not ILC1 or ILC3, expressed DNAM-1 [160]. Collectively, these studies have revealed a great heterogeneity in the expression of TIGIT, CD96 and DNAM-1 by ILC, extending initial observations made on NK cells and T cells. However, the role and function of these receptors in non-NK cell ILC remains to be fully elucidated.

### 6.5. ICOS—ICOSL

ICOS is a member of the CD28 superfamily [225], and is highly expressed on activated and regulatory T cells [225,226,227], while its ligand (ICOS-L) is expressed by both immune and non-immune cell types [228,229,230]. ICOS promotes T cell function and survival as well as Th2 responses [226,231,232,233]. Similar to T cells, ILC2 express high levels of ICOS, which influences their function and regulates ILC2-dependent immunity [186,187,188,189] (Figure 2). In mice, ICOS deficiency resulted in reduced numbers of ILC2 in the lung and small intestine, in part due to impaired ILC2 proliferation and survival. The absence of ICOS also led to decreased ILC2-derived IL-13 production [187,188]. Mechanistically, ICOS-deficient ILC2 showed decreased expression of the pro-survival molecule BCL2 upon activation [187]. In vivo, loss of ICOS abrogated ILC2-dependent lung inflammation following intranasal IL-33 injections [187]. ILC2 also express ICOS-L [186,187] which promotes ILC2-derived IL-13 expression and survival [187]. In addition, ICOS-L-expressing ILC2 support the accumulation of ICOS-expressing Tregs in visceral adipose tissue [186]. In vitro blockade of the ICOS-ICOS-L signalling pathway in ILC2-Treg co-cultures specifically reduced expansion of Tregs but not naïve CD4 T cells [186], indicating that ICOS-L-expressing ILC2 sustain Treg cell homeostasis. Conversely, induced inflammatory Tregs (iTregs) mediate ILC2 suppression through the ICOS-ICOS-L pathway [234]. Reduced ILC2-derived IL-5 and IL-13 production were detected in ILC2-iTregs co-cultures. In addition, adoptive transfer of iTregs along with IL-33 administration or *Alternaria* extract inoculation alleviated ILC2-dependent lung inflammation, a finding that was further validated using syngeneic human ILC2 and iTreg transfer in IL-33-treated NSG mice [234]. In humans, ILC2 express both ICOS and ICOS-L, which promote ILC2-dependent inflammation [187]. While mainly associated with ILC2, *ICOS* is also expressed by circulating human ILC1. Its ligand, *ICOSLG* is expressed by both circulating ILC1 and ILCp [111]. However, the role and function of the ICOS–ICOSL signalling pathway in these ILC subsets remains to be determined. Collectively, this signalling pathway act as a key regulator of ILC2 function and ILC2-dependent inflammation, but whether similar mechanisms occur in tumors and how it impacts anti-tumor immunity has yet to be defined.

### 6.6. LAG3

LAG-3 (CD223) is an inhibitory molecule expressed on activated T cells. Persistent antigenic stimulation induces the upregulation of LAG-3 on T cells, which is associated with dysregulated T cell responses [112]. The extracellular domain of LAG-3 resembles the co-receptor CD4, and binds to MHC-Class II with higher affinities than CD4 [112]. Additional ligands have been described and include galectin-3, the lectin LSECtin and the fibrinogen-related protein (FGL)-1 [112]. Interactions between LAG-3 and its ligands impair anti-tumor immunity [112]. Besides conventional T cells, LAG-3 is expressed by multiple other immune populations, including regulatory T cells, B cells, plasmacytoid dendritic cells, NK cells and ILC1 [111,112,161] (Figure 2). However, little is known about the function of LAG-3 on non-T cell immune cells, particularly ILC. Recent findings suggest that T cells and NK cells upregulated LAG-3 in chronic lymphocytic leukaemia [148]. The use of an anti-LAG-3 blocking antibody in vitro increased T cell and NK cell proliferation and enhanced T cell TNF-α, IL-2 and IFN-γ production [148], indicating that anti-LAG-3 antibody may potentially act on both innate and adaptive immune lymphocytes. Chronic stimulation of NK cells through NKG2C, NKp30 or NKG2D was shown to induce PD-1 and LAG-3 upregulation [144]. LAG-3^+^ NK cells display reduced IFN-γ expression [144]. In mice, reduced NK cell cytotoxicity linked to the deletion of LAG-3 has also been reported [145]. However, this finding was not confirmed using anti-LAG-3 blocking antibodies [146]. NK cell stimulation with IFN-α induced LAG-3 upregulation, together with increased expression of TIM-3 and PD-1 [147]. Interestingly, in vitro blockade of LAG-3 using antibodies increased NK-cell-derived cytokine expression without modifying NK cell cytotoxicity potential [147]. In mice, tumor-infiltrating ILC1-like cells transdifferentiated from NK cells downstream of TGF-β signalling expressed higher levels of LAG-3 than NK cells [161]. Regarding LAG-3 expression in non-NK cell ILC, the transcriptional analysis of circulating ILC isolated from healthy donors has revealed that ILC1, but not ILC2 or ILC3, express *LAG3* [111]. Collectively, these findings indicate that stimulated ILC may express LAG-3, particularly NK cells and ILC1. It remains to be determined whether tumor-infiltrating ILC express LAG-3 and, if so, what the consequences of LAG-3 expression are on ILC function, patient prognosis and therapeutic responses.

### 6.7. CD137/4-1BB

CD137/4-1BB stimulation using agonist monoclonal antibodies limit tumor growth [235]. Besides T cells, expression of CD137 is observed on dendritic cells, eosinophils, monocytes, mast cells, NKT cells and NK cells [235] (Figure 2). Mouse ILC2 residing in the large intestine, and to a lesser extent in the small intestine, expressed CD137 intracellularly. Interestingly, CD137 was preferentially expressed by activated IL-5-expressing ILC2 [42]. In humans, lung ILC2, but not blood or tonsil ILC2, expressed *TNFRSF9* [34,111]. In accordance, ILC2 in bronchiolar alveolar lavage samples obtained after allergen challenge increased *CD137* expression compared to blood ILC2 [190]. However, in another study, no changes in CD137 expression were found on in vitro stimulated ILC2 despite multiple culture conditions from both healthy donors or allergic patients [180]. While additional studies are warranted, evidence to date predominantly supports that tissue resident ILC2 may express CD137; yet the conditions of when and how CD137 is induced on ILC2, as well as how these impact various disease processes, remain unknown.

### 6.8. KLRG1

KLRG1, a C-type lectin, is expressed by many immune cell types, including T cells, NK cells and ILC2 [150,169,170,174,175,176] (Figure 2). KLRG1 expression together with CD11b upregulation on peripheral NK cells identifies more mature cells with increased capacities to produce pro-inflammatory cytokines [150]. High KLRG1 expression was associated with reduced proliferative capacity and increased apoptosis of NK cells [151]. In addition, in vitro cultured KLRG1^+^ NK cells have reduced cytotoxic activity against KLRG1 ligand-expressing target cells, which can be reversed by using a blocking antibody [149]. The deficiency in KLRG1 expression in mice does not impact T cell or NK cell frequencies nor NK cell maturation at steady state [152]. However, a reduced B16 melanoma cell line metastatic burden was observed in KLRG1-deficient mice compared with wildtype controls [152], confirming the inhibitory function of KLRG1 in the anti-tumor immune response. The treatment of tumor-bearing mice with a combination of anti-PD-1 and anti-KLRG1 antibodies further improved the anti-tumor response and was associated with increased intratumor T and NK cell accumulation compared to single treatments [152]. KLRG1 expression on ILC2 identifies an activated subset following IL-25 or IL-33 stimulation [46,170,173]. KLRG1 deficiency does not impact lung ILC2 frequency nor their expression of the IL-33 receptor, ST2 [177]. However, in a competitive mixed bone marrow chimeric setting, KLRG1 expression significantly reduced the ability of ILC2 to accumulate in the lungs following IL-33 injection [177], indicating that KLRG1 negatively regulates ILC2 function in this context. However, no difference was observed for lung ILC2 function in response to papain challenge between KLRG1-sufficient and deficient mice [177]. Circulating, tonsil and lung-resident ILC2 displayed significantly higher levels of *KLRG1* than other identified ILC subsets [34]. KLRG1 expression was also recently shown to mark ILC2_10_, which correlated with response to allergy immunotherapies and was associated with the attenuation of T helper responses and maintenance of epithelial cell integrity [236]. While KLRG1’s expression on ILC in the context of cancer is relatively unexplored, KLRG1-expressing ILC2 are found within the circulation of healthy donors, and the non-tumoral tissues, lung and colorectal tumors of cancer patients [40]. Future studies should delineate the function of KLRG1 on ILC2-derived cytokine production, proliferation and implications for anti-tumor immunity.

### 6.9. GITR—GITRL

GITR, also known as TNFRSF18, is a member of the TNF superfamily [237]. GITR is expressed by multiple immune cell types, including innate and adaptive immune cells, and its expression is further enhanced upon activation [237]. Binding to its ligand, GITRL, or using agonistic antibodies, results in effector T cell activation, but also suppression of inhibitory function of Tregs [237]. This has prompted the development of agonistic antibodies which elicited potent anti-tumor responses in pre-clinical mouse models [237]. NK cells and other ILC subsets also express GITR (Figure 2). In NK cells, GITR surprisingly acts as an inhibitory molecule, dampening NK cell activity and antitumor function [153,154,155,156,157]. The expression of GITR has been reported on murine ILC1 and is upregulated following influenza infection [168]. Like NK cells, GITR inhibits ILC1 activity, impairing ILC1-mediated IFN-γ production [168]. ILC2 express high levels of GITR, which can be further increased following *Alternia*-induced lung inflammation [191,192]. ILC2 GITR expression was correlated with higher levels of IL-5 and IL-13 [191,192] and its engagement using an agonistic antibody promoted ILC2-derived type 2 cytokines associated with increased protection against metabolic disorders induced by high-fat diet treatment or type 2 diabetes [192]. Interestingly, GITRL and ICOSL expression by mouse ILC2 was shown to be important for the resolution of inflammation in a mouse model of rheumatoid arthritis. Here, ILC2 supported Treg-suppressive functions, which were required to dampen Th17-mediated autoimmunity and resolve joint inflammation [238]. In humans, GITR is expressed on ILC2 and GITR ligation promotes ILC2-derived IL-5, IL-13, GM-CSF, IL-9 and IL-8 production [192]. GITR may also regulate ILC3. Tonsil NKp46^+^ ILC, NKp44^+^ ILC3 and NKp44^−^ ILC3/ILCp expressed significantly higher levels of *TNFRSF18* than circulating ILC2 and KLRG1^+^ ILC [58]. These findings collectively indicate that all ILC subsets express GITR, yet GITR stimulation can have differential effects depending on the ILC subset. While GITR inhibits NK cell and ILC1 activity, GITR stimulates ILC2 function. The role and function of GITR expression on non-NK cell ILC in tumor immunity remains to be determined.

### 6.10. BTLA—HVEM

BTLA is expressed on splenic B and T cells, and negatively regulates immune responses [239]. BTLA binding to herpesvirus entry mediator (HVEM) induces BTLA phosphorylation, its association with SHP-2 and the repression of antigen-driven T cell proliferation. Besides its expression on adaptive immune cells, BTLA expression has been described on NK cells [159] (Figure 2), NKT cells and myeloid cells [239]. In chronic lymphocytic leukaemia, BTLA expression was elevated on NK cells in comparison with healthy donors [159]. BTLA engagement reduced NK cell-derived IFN-γ production, whereas the blockade of this receptor increased IFN-γ expression and the killing capacities of NK cells [159]. More importantly, high levels of BTLA on NK cells were associated with reduced patient survival [159]. Recently, BTLA gene expression has also been detected on non-NK cell ILC. Circulating ILC1, but not ILC2 or ILCp, expressed *BTLA* to a similar level to that of Th1 cells [111]. Furthermore, circulating ILCp expressed high levels of *TNFRSF14* (HVEM) [111]. Validating the expression of BTLA and HVEM at the protein level, as well as studies addressing how these molecules impact ILC function are needed. 

### 6.11. OX40—OX40L

OX40, a member of the TNF receptor superfamily, is expressed by activated T cells, particularly helper CD4 T cells and Tregs. OX40 sustains T cell proliferation, survival and promotes CD4 T cell memory while it suppresses Treg activity [240]. Although OX40 is expressed on T cells, its ligand, OX40L (CD252), has been found on antigen-presenting cells such as dendritic cells and B cells, influencing T cell activation and function [240]. Beyond these well-described antigen-presenting cells, ILC also modulate the adaptive immune response through the expression of MHC class II molecules and antigen presentation [14]. Both ILC2 and ILC3 express OX40L, which regulates adaptive immune cell function and responses to infections and allergens [193,194,195,196,197,198] (Figure 2). In *Il7r^−/−^* lymphoid-deficient mice, adoptive transfer of lung ILC2 and CD4 T cells was required to generate a potent type 2 immune response [195]. In vitro analyses of CD4 T cell and ILC2 co-cultures have revealed that the OX40/OX40L signalling pathway was critical to CD4 T cell polarization towards a Th2 profile [195]. Lung ILC2 expressed high levels of OX40L upon papain or IL-33 intranasal administrations and specifically promoted the expansion of OX40-expressing Th2 and Treg cells [193]. *Tnfsf4* deletion in ILC2, but not in CD11c-, RORγt- or CD4-expressing cells impaired adaptive immune cell responses in response to IL-33 injection [193], demonstrating a requirement for ILC2 in the generation of Th2 and Treg responses in this setting. In addition, ILC2-expressed OX40L was critical for the generation of anti-helminth responses and type 2 immunity to allergens [193]. Mice infected with the respiratory syncytial virus displayed increased CD4 T cells in the lungs, an effect dependent on ILC2 [194]. This adaptive immune cell expansion was accompanied by enhanced OX40 and OX40L expression on lung CD4 T cells and ILC2, respectively, and the OX40/OX40L interaction promoted CD4 T cell cytokine production [194]. These findings suggest that ILC2-expressed OX40 is critical to drive OX40L-expressing T helper cell expansion, type-2 polarization and function, but whether similar regulation occurs in tumors is yet to be determined. The expression of OX40L in LTi cells is required for the maintenance of CD4 T cell memory within secondary lymphoid organs [197]. Furthermore, ILC3 OX40L expression plays a critical role in maintaining the homeostasis of the intestinal barrier [196,198]. Intestinal ILC3 constitutively expressed OX40L, but its level is further increased upon inflammation [196]. Particularly, the microbiota and intestinal inflammation promoted CX3CR1^+^ mononuclear macrophage TNF-like ligand 1A (TL1A) secretion, driving ILC3 OX40L upregulation and IL-22 expression [198]. OX40L-expressing ILC3 promoted conventional and regulatory CD4 T cell proliferation and function [196,198]. In cryptopatches, ILC3 and Tregs interacted with each other, and the transfer of OX40L-deficient ILC3 together with Tregs in *Tnfsf4^−/−^Rag1^−/−^* mice impaired the expansion of Tregs [196]. Furthermore, deletion of OX40L in ILC3 compromised the maintenance of OT-II cells and their activation and effector function, reducing the susceptibility of mice to DSS-mediated intestinal inflammation [198]. Collectively, these results indicate that OX40L-expressing ILC3 regulates intestinal inflammation and adaptive immune response, deserving further investigation of this pathway in intestinal carcinogenesis. In humans, circulating ILCp expressed high levels of *TNFRSF4*, supporting previous findings which showed that circulating NKp46^+^ ILC, NKp44^−^ ILC3 (potentially ILCp) expressed high levels of *TNFRSF4* [58,111]. Additionally, *TNFSF4* was found specifically expressed in tonsils, lung and colon ILC3 but not in other ILC subsets [34], supporting previous observation showing that intestinal ILC3 isolated from patients with Crohn’s disease expressed OX40L [198]. These data suggest that OX40L expression impacts ILC3 function, with implications for intestinal homeostasis, inflammation and tumorigenesis [204].

## 7. Conclusions

The diversity of ILC in tissues, their capacity to migrate to distant organs upon tissue insult and the expression of immune checkpoint molecules at their surface identify ILC as key orchestrator of immune responses, offering a rational targeting, yet to be investigated in clinical trials. The term “immune checkpoints” is usually used to describe cellular surface molecules expressed by immune cells which, upon binding to their ligands, suppress the immune response, with PD-1 and CTLA-4 seen as the prototypical family members. However, IC include a vast array of stimulatory and inhibitory receptors and ligands, cytokine signalling pathways, transcription factors, metabolic pathways and intracellular inhibitory proteins, which together modulate ILC activity and function. Integration of these signals within distinct tissue microenvironments certainly influences tissue homeostasis, pathogenesis and anti-tumor responses. Whether these specificities extend to IC expression, subsequently impacting anti-tumor immunity and therapeutic responses, remain to be fully determined. In addition, it remains to be determined whether these IC that have been shown to be expressed by ILC play a similar role or alternate function to what has been documented for T cells. Furthermore, a given IC can both promote or inhibit ILC function depending on the cell type. Thus, the use of IC inhibitors can drive unwanted immune consequences affecting therapeutic efficacy and the patient’s clinical prognosis. Currently, this gap in our knowledge directly impacts our ability to consider the expression of IC on ILC in the design of future clinical trials. A better understanding of these signalling pathways and their consequences on ILC function in tumors are urgently needed to offer additional targeting opportunities in cancer. Ideally innate lymphocyte responses would be modulated to work in parallel with anti-tumor T cells to promote potent and long-lasting anti-tumor immunity.

## Figures and Tables

**Table 1:** Immune checkpoint expression and function according to ILC subsets.

ILC Subset	Checkpoint Marker	Ligands	Mouse	Human	Function	Context of Expression	References
NK cells	PD-1	PD-L1/2	Yes	Yes	Negative regulation. Reduced NK cell cytokine production and cytotoxicity resulting in tumor escape.	Induced upon activation by viral infections including MCMV, HCMV, HIV and HCV as well as tumor microenvironment including ovarian carcinoma, Kaposi sarcoma, Hodgkin lymphoma, pleural effusions of primary and metastatic tumors, digestive, lung and breast cancers.	[114,115,116,117,118,119,120,121,122,123,124]
	TIM-3	Galectin-9, phosphatidylserine, HMGB1, Caecam-1	Yes	Yes	Negative regulation. Impaired NK cell-derived IFN-γ and TNF-α expression as well as perforin production and cytotoxicity.	Expressed at steady state (CD56^dim^ NK cells). TIM-3 is expressed in NK cells upon stimulation with IL-2, IL-12+IL-18 or IL-15 and cancers including gastrointestinal stromal tumors, gastric cancer, oesophageal cancer, melanoma, colorectal cancer, anaplastic thyroid cancer, bladder cancer, hepatocellular carcinoma and lung adenocarcinoma.	[125,126,127,128,129,130,131,132,133,134,135,136]
	TIGIT	CD155 CD112	Yes	Yes	Negative regulation. Impaired NK cell activity.	Expressed at steady state in circulation and tumor microenvironment including in melanoma and colorectal cancer.	[137,138,139,140,141,142]
	LAG-3	MHC-II, galectin-3, LSECtin, FGL-1		Yes	Negative regulation. Reduced IFN-γ expression and may inhibit NK cell function.	Chronic stimulation through NKG2C, NKp30 or NKG2D and stimulation with IFN-α as well as chronic lymphocytic leukaemia.	[143,144,145,146,147,148]
	KLRG1	E-, N, R-Cadherin	Yes	Yes	Negative regulation. Reduced NK cell cytotoxicity, proliferation and increased apoptosis.	Klrg1^−/−^ NK cells have increased capacities to produce pro-inflammatory cytokines in tumor microenvironment	[149,150,151,152]
	GITR	GITRL	Yes	Yes	Negative regulation. Dampened NK cell function, proliferation, and survival.	Reduced anti-tumor activities	[153,154,155,156,157]
	BTLA	HVEM		Yes	Negative regulation. Reduced NK cell IFN-γ expression and killing capacity.	Chronic lymphocytic leukaemia	[158,159]
	CTLA-4	CD80, CD86	Yes	Yes	Not determined	A fraction of circulating NK cells in melanoma patients.	[160]
ILC1	PD-1	PD-L1/2		Yes	Not determined	Tumor microenvironment including non-small cell lung cancer, breast and gastrointestinal tumors.	[124,161,162,163]
	CTLA-4	CD80, CD86	Yes	Yes	Not determined	Tumor microenvironment including melanoma, hepatocellular carcinoma, breast and gastrointestinal cancer.	[49,111,124,160,161,164,165,166,167]
	TIGIT	CD155 CD112	Yes		May negatively regulate ILC1 function.	Tumor microenvironment	[161]
	LAG-3	MHC-II, galectin-3, LSECtin, FGL-1	Yes	Yes	Not determined	Tumor microenvironment	[111,161]
	KLRG1	E-, N, R-Cadherin		Yes	Not determined	Tumor microenvironment including non-small cell lung cancer, breast and gastrointestinal tumors.	[124]
	GITR	GITRL	Yes		Negative regulation. Inhibited ILC1 IFN-γ production and activity.	Expressed at steady state and enhanced upon activation following influenza infection.	[168]
ILC2	KLRG1	E-, N, R-Cadherin	Yes	Yes	Negative regulation. May negatively regulate ILC2 accumulation and function in the lungs following IL-33 injection.	During steady state on iILC2 and upon IL-25 or IL-33 activation on nILC2.	[46,149,150,169,170,171,172,173,174,175,176,177]
	PD-1	PD-L1/2	Yes	Yes	Negative regulation. Regulation of ILC2 metabolism and STAT5 phosphorylation resulting in decreased cytokine expression and proliferation.	Lowly expressed at steady state and is further increased upon IL-33 and γc cytokines stimulation in contexts including influenza infection, papain challenge and cancer including breast, gastrointestinal, colorectal, melanoma, non-small lung and pancreatic adenocarcinoma cancers.	[44,46,49,124,162,163,170,178,179,180,181]
	PD-L1	PD-1	Yes		Promoted Th2 polarization and type 2 inflammatory responses.	*Nippostrongylus brasiliensis* infection and retinoic acid stimulation.	[111,170,182] [183]
	CTLA-4	CD80, CD86	Yes	Yes	Negative regulation. May negatively regulate ILC2 maintenance and may bestow immunosuppressive properties on ILC2.	Retinoic acid stimulation and tumor microenvironment including hepatocellular carcinoma, breast, and gastrointestinal cancer.	[49,124,183,184]
	ICOS	ICOS-L	Yes	Yes	Positive regulation. Promoted lung and small intestine ILC2 accumulation, cytokine expression, survival, and proliferation.	Notably expressed on lung and skin ILC2.	[185,186,187,188,189]
	CD137/ 4-1BB	CD137L/ 4-1BBL	Yes	Yes	May modulate ILC2 effector function.	Expressed in mouse large and small intestine ILC2.	[34,42,111,190]
	GITR	GITRL	Yes	Yes	Positive regulation. Promoted ILC2 cytokine expression and function.	Expressed at steady state and has an increased expression upon activation including *Alternia*-induced lung inflammation.	[191,192]
	OX40L	OX40	Yes		Promoted Th2 and Treg cell responses critical to anti-helminth and allergic type 2 immunity.	Upon papain or IL-33 stimulation, helminth infection, allergic reactions, and respiratory syncytial virus infection.	[193,194,195,196,197,198]
ILC3	PD-1	PD-L1/2	Yes	Yes	Negative regulation. Inhibited ILC3 function.	Mouse intestine, human decidua and tumors including breast and gastrointestinal tumors.	[49,121,124,162,199]
	TIM-3	Galectin-9, phosphatidylserine, HMGB1, Caecam-1		Yes	Negative regulation. May inhibit ILC3 function.	Human decidua	[199,200,201,202,203]
	OX40L	OX40	Yes	Yes	LTi cell expression is required for CD4^+^ T cell memory maintenance within secondary lymphoid organs. ILC3 maintains intestinal barrier homeostasis and promotes conventional and regulatory CD4^+^ T cell maintenance, proliferation, and function.	Microbiota and intestinal inflammation promote mononuclear macrophages TL1A secretion, which drives ILC3 OX40L expression. Hence, intestinal ILC3 constitutively express OX40L and the level of expression is increased upon inflammation such as Crohn’s disease.	[196,197,198,204]
	CTLA-4	CD80, CD86	Yes		May negatively regulate ILC3 maintenance.	Hepatocellular carcinoma	[49,184]
